# Thomson’s Conspectus of the PharmacopœIas of the United States and Great Britain

**Published:** 1850-03

**Authors:** 


### ARTICLE II.

*A Conspectus of the Pharmacopœias of the London, Edinburgh, and Dublin Colleges of Physicians, and of the United States Pharmacopœia; being a Practical Compendium of Materia Medica and Pharmacy*—By ANTHONY TODD THOMSON, M.D. F.L.S., Fellow of the Royal College of Physicians, Professor of Materia Medica and Therapeutics in Univ. Coll. London, etc., etc., Edited by CHARLES A. LEE, Professor of General Pathology and Mat. Med. in Geneva Medical College. Samuel S. Wood, New-York: pp. 300: 16 mo: (From the Publisher. For sale by Jos. Keen, & Bro., Chicago, Ill.)

This is a small book with a large title; and constitutes a very convenient compend for reference as to the origin, use, properties, dose, toxicological effects, etc., etc., of the various articles of the Materia Medica. We recommend it as such to those of our readers who are not already in possession of it.

